# The influence of large-diameter multifocal contact lens on ocular surface, visual quality, and visual function for presbyopic adults with dry eye syndromes

**DOI:** 10.1038/s41598-023-46732-6

**Published:** 2023-11-09

**Authors:** Ching-Jen Hsiao, Hsiao-Ching Tung, Chuen‐Lin Tien, Yu-Wen Chang, Ching-Ying Cheng

**Affiliations:** 1 Department of Optometry, Chung Shan Medical University, Taichung, Taiwan; 2 Department of Ophthalmology, Chung Shan Medical University Hospital, Taichung, Taiwan; 3 Dr. Don’s Eye Clinic, Taipei, Taiwan; 4 Department of Electrical Engineering, Feng Chia University, Taichung, Taiwan

**Keywords:** Health care, Medical research

## Abstract

This study investigated the influence of large-diameter multifocal contact lenses on the ocular surface, visual quality, and visual function for presbyopic adults with dry eye syndromes. The study enrolled 40–55-year-old adults with presbyopia and dry eye syndromes (DES). The subjects were randomly assigned to three groups wearing different designs of contact lenses (Proclear, SMR, and Optimum) for 6–8 h a day for two weeks. Ocular surface health, tear quality, visual quality, and visual function were measured before and after lens wear. No significant difference was observed across all three groups for the amount of conjunctival redness, blink frequency (lens on), and stereopsis vision before and after wearing. Although there seemed to be a significant declining trend for corneal staining and limbal redness, non-invasive tear break-up time (TBUT), and lipid layer thickness while lens wear, the measured values were all within the normal range. Vice-versa after lens removal, results also showed significant improvement on lipid layer thickness, blink frequency (lens off), and contact TBUT. A significant improvement was observed in the modulation transfer function (MTF) of the total area ratio after wearing contact lenses. In contrast, the MTF of the high-order aberration area ratio resulting from lens wear was lower than that of the baseline measurement. There are also significant improvements observed for SMR and Optimum regarding near visual acuity, near point of accommodation, and the subjective questionnaire (OSDI and VBP) scores. Although it is difficult to avoid a specific negative impact on the ocular surface and tear film, visual function and visual quality can still be positively improved, especially shown on larger diameter and distance-center designed multifocal contact lenses.

## Introduction

Contact lens wear is one effective option for correcting ametropia^1–4^. However, the majority of individuals opt for contact lenses to correct their refractive errors before reaching the age of 30 to 34. Beyond this age range, changes in accommodation^5^ and physiological mechanisms, such as a reduction in the number and size of goblet cells, as well as decreased lacrimal gland secretion, can lead to conjunctival alterations and dryness of the eyes. This, in turn, can lead to discomfort and may even increase the risk of infection when wearing contact lenses^6,7^.

Dry eyes, a common condition seen in ophthalmology clinics^8^, are now manifesting at increasingly younger ages^9–12^. The frequent use of digital devices often reduces the rate of eye blinking, prolonging the exposure of the eye surface to the air, disrupting the delicate balance between tear evaporation and tear film replenishment^13^^.^ Long-term users of digital products, approximately 64% to 90% of them, are susceptible to experiencing symptoms associated with Computer Vision Syndrome (CVS), which commonly include headache, shoulder and neck pain, dry eyes, eye-surface congestion, blurred vision, diplopia, and eye fatigue^14,15^. CVS symptoms are closely related to dry eye symptoms; approximately 75% of people with CVS suffer from dry eyes or dry eye syndromes^16,17^, high-order aberration, visual fluctuation, glare, poor contrast sensitivity and visual quality^18,19^.

Many contact lenses have been designed and developed using different materials and optical designs to correct presbyopia and have also been designed based on wearing needs to improve visual quality and visual comfort when wearing contact lenses for extended periods^4,20–22^. According to previous studies, rigid contact lenses (RGP) offer better ocular health due to increased tear exchange and higher oxygen permeability than soft contact lenses (SCL)^23^. Recent research has been exploring large-diameter RGPs lenses^24–27^, with a particular focus on their impacts on DES, tear film quality, visual quality, visual function, and binocular accommodation.

Large-diameter RGPs, also known as scleral contact lenses, are defined with a diameter > 12.5 mm. It is designed to only have direct contact with the ocular surface at the bulbar conjunctiva and thus create corneal vaults filled with preservative-free (PF) saline solution. The PF saline solution-filled space between corneal surface and the rear surface of the lens provides a stable tear film^24,28,29^. Due to the breathable material and design, large-diameter RGPs are often used to treat keratoconus (KCN), high astigmatism that cannot be corrected with small-diameter rigid lenses, ocular surface diseases, and simple refractive correction^30–32^. They are also a preferred choice over soft contact lenses for their ability to provide tear storage between the lens and the cornea, offering relief from discomfort associated with dry eye syndrome (DES)^25,33^.

Large-diameter multifocal contact lenses, whether soft or rigid, are designed for simultaneous vision with minimal movement to achieve a translating effect. These multifocal contact lenses can further be categorized as center-near (CN) for improved near vision with a trade-off of blurrier distant vision, and center-distant (CD) for the vice versa^34^. The purpose of this study was to analyze the impact of wearing different types of large-diameter multifocal contact lenses on the ocular surface, tear film, and visual quality in presbyopic adults with dry eye syndrome (DES).

## Materials and methods

### Study design

A mixed two-factor design (3 groups × 4 measurements) experimental study was conducted from November 18, 2020, to May 30, 2021, at Chung Shan Medical University, Taichung, Taiwan. This experimental study protocol was reviewed and approved by the institutional review board of Chung Shan Medical University Hospital (approval no. CS2-20,089), and the study strictly adhered to the research ethics specifications of the Declaration of Helsinki. To ensure accurate measurement results, possible confounding factors such as laboratory brightness, visual target distance, and subjective differences in measurement tools and equipment operators were controlled (STROBE guidelines for reporting the manuscript^35,36^).

### 2.2. Research subjects

After the investigators thoroughly described the study content and procedure, subjects voluntarily decided to participate in the study and signed the informed consent form. The Ocular Surface Disease Index (OSDI) and Dry Eye Questionnaire (DEQ-5) were administered for preliminary screening. Subjects with OSDI scores ≥ 13 and 6 < points DEQ-5 scores < 12 were included in the study, it indicated that subjects who met the screening criteria all displayed subjective dry eye symptoms.

At first, 68 presbyopic adults aged 40–55 years completed the OSDI and DEQ-5 questionnaires. Of these, 60 subjects were enrolled after screening. The inclusion criteria for the study were refractive errors of myopia ≤ 8.00 D, astigmatism ≤ 1.75 D, and near ADD ≥ 0.75 D. Subjects who had undergone at least one eye surgery, eye-related diseases, ocular surface dysfunction, systemic immune-related diseases, long-term usage of eye drops, or could not wear contact lenses were excluded. Of the 60 people included after the initial screening, 18 were subsequently excluded: 5 subjects had refractive errors that did not meet the criteria, 5 were unable to adapt to wearing contact lenses, and 8 did not cooperate with the follow-up schedule. Finally, 42 subjects (12 Proclear Lens: 46.33 ± 3.75; 12 SMR Lens: 46.83 ± 5.06; and 18 Optimum Lens: 45.78 ± 3.17; 12 men: 46.67 ± 5.14 and 30 women: 46.07 ± 3.33) with a mean age of 46.24 ± 3.88 years participated and were randomly assigned to different lens group, If the assigned group is not suitable for the subject, it is handled by dropping them out. One-way analysis of variance (ANOVA) showed no significant differences in age, OSDI, DEQ-5 score, or refractive errors among three lens groups (Table 1).

**Table 1:** General information comparison of lens groups (Mean ± Standard Deviation).

Group	N	Age M ± SD	OSDI M ± SD	DEQ-5 M ± SD	Right Sph(D) M ± SD	Right Cyl(D) M ± SD	Left Sph(D) M ± SD	Left Cyl(D) M ± SD
Proclear (Soft CL)	12	46.33 ± 3.75	18.33 ± 4.36	9.42 ± 3.18	− 5.33 ± 2.63	− 0.48 ± 0.21	− 5.38 ± 2.28	− 0.52 ± 0.46
SMR (ArtMost, Soft CL)	12	46.83 ± 5.06	17.42 ± 1.78	8.83 ± 1.53	− 6.71 ± 1.22	− 0.67 ± 0.63	− 6.54 ± 1.43	− 0.67 ± 0.56
Optimum (Rigid CL)	18	45.78 ± 3.17	16.44 ± 2.28	9.00 ± 2.38	− 5.31 ± 2.02	− 0.83 ± 0.52	− 5.33 ± 2.02	− 0.86 ± 0.43
ANOVA F and *P*-value		*F* = 0.262 *p* = 0.771	*F* = 1.532 *p* = 0.229	*F* = 1.263 *p* = 0.294	*F* = 2.002 *p* = 0.149	*F* = 1.828 *p* = 0.174	*F* = 1.590 *p* = 0.217	*F* = 1.874 *p* = 0.167

*P* < 0.05.

The sample size of this study was determined using G*Power analysis, with an effect size of *f* = 0.8, α = 0.05, power (1-*β*) = 0.95, and number of groups = 3. The calculated results of the total sample size were 30. The number of participants who completed the study (42) exceeded the sample size required (30); the power appeared to be adequate after recalculation and adjustment (effect size *f* = 0.8, α = 0.05, power (1 − β) = 0.984).

### Research materials

#### Contact lenses

Three types of multifocal contact lenses were used to compare the dependency variances between different optical or different material designs (Table 2): Lens 1: CooperVision Proclear® 1-day multifocal Lens (Proclear); Lens 2: ArtMost Aspherical progressive soft contact lens (SMR); and Lens 3: Optimum GP rigid gas-permeable contact lenses (Optimum). All the contact lenses used in this study had passed FDA approval for a broad range of indications and contact lens modalities. Table 2 lists the contact lens design and specification parameters. All three contact lenses have multifocal designs; lens material and lens design are enhanced for eye parts prone to dryness. All subjects were asked to wear the contact lenses for 6–8 h a day. To avoid any potential sources of bias, each test was performed by the same optometrist. All participants were invited to undergo baseline (wear multifocal spectacles) examination, as well as fellow up on D1, W1, W2 after wearing contact lenses.

**Table 2:** Contact lens design and specification parameters.

Contact lens	Optical design	Material	Basic curve (mm)	Diameter (mm)	Water content (%)	Oxygen permeability(cm^2^/s) (ml O_2_/mL × mm Hg)
Proclear (Soft CL)	Aspherical Progressive (Near–center)	Omafilcon A	8.7	14.2	60%	21 × 10^−11^
SMR (ArtMost, Soft CL)	Aspherical Progressive (Distance–center)	Ocufilcon D	9.0	14.4	55%	19.6 × 10^−11^
Optimum (Rigid CL)	Aspherical Progressive (Distance–center)	Optimum Extra (Raflufocon D)	6.49–8.43	15.5	–	100 × 10^−11^

The process of fitting begins with an initial assessment, which includes evaluating eye health, visual acuity, and specific refractive errors, as well as patient's medical history. Subsequently, a trial lens fitting assessment are performed, which involve checking for signs of decentration, excessive movement, or lens tightness. The central cornea and limbus should not experience compression, and a certain tear thickness should be ensured. Initially, the central area generally needs to maintain a tear thickness of about 300 µm. The initial position of the landing zone should be located at approximately halfway between the corneal edge and the lens edge, and it should not exert excessive pressure on the cornea. After wearing the trial lenses for 30 min, the central tear thickness should still be around 150–200 µm. Only after determining the appropriate trial lenses should a refraction check be performed. Subsequent follow-up appointments, training, education, and regular monitoring were required.

#### Measurement

Measurements in the study included: (1) ocular surface; (2) tear quality; (3) visual quality; and (4) visual function. Each examination was performed on the baseline (BL), day 1, week 1, and week 2. In addition to the baseline measurement data (wearing habitual multi-focal spectacles) without participants wearing contact lenses before the experiment, some exams could only be measured after removing the lenses (ocular surface redness, corneal fluorescence staining, and contact TBUT), while others could be performed with or without participants wearing contact lenses, including NI-TBUT, lipid layer, and blink frequency. Visual quality included blinking frequency and modulation transfer function (MTF), as well as the analysis of the subjective questionnaire survey. Blinking frequency was also recorded while wearing and removing contact lenses.

A Topcon VT-10 phoropter (Topcon, Tokyo, Japan), View-M digital visual acuity chart (Quan Chin Industrial Co., Taiwan, 6 M in a bright room), and a TMV near-point card (Brighten Optix Co., Taiwan, 40 cm) were used to measure subjective refraction and visual acuity. Further examinations conducted for this study involved the following four stages: (1) ocular surface evaluation (Topcon SL-D701 slit lamp), including quantifying the redness of the conjunctiva or limbus (Efron Grading Scales for Contact Lens Complications, score 0–4), and corneal fluorescence staining (standard Oxford scale, score 0–5); (2) tear quality, including contact tear-film break-up time (TBUT), non-contact tear-film break-up time (NI-TBUT), and lipid-layer thickness (SBM ICP Tearscope); (3) visual quality examination, including blink frequency, modulation transfer function (Nidek OPD Scan 3, Tokyo, Japan)^37^, and dry-eye-related questionnaires (Dry Eye Questionnaire, DEQ-5 and Ocular Surface Disease Index, OSDI); (4) visual function measurement, including near visual acuity, stereo visual acuity (Butterfly Stereo Acuity test), and near point of accommodation (Royal Air Force Ruler, RAF; and Push-up method).

### Data analysis and statistical analysis

All data were obtained and analyzed using SPSS 22.0 statistical software (IBM, Armonk, NY, USA). A value of ***p*** < 0.05 was considered statistically significant. Repeat-measurement analysis, two-way ANOVA and simple main effects analysis were performed. Because the participants were all healthy subjects, there was no significant difference between left and right eyes in terms of refractive errors; the results only showed the left eye parameters.

### Institutional review board statement

The study was conducted according to the guidelines of the Declaration of Helsinki and approved by the Ethics Committee of Chung Shan Medical University Hospital (Taichung, Taiwan) (Approval Number: CS2-20089). Informed written consent was obtained from all individual participants included in the study.

### Informed consent

Patients signed informed written consent regarding the publication of their data or photographs.

## Results

### Ocular surface

**Figure 1:**
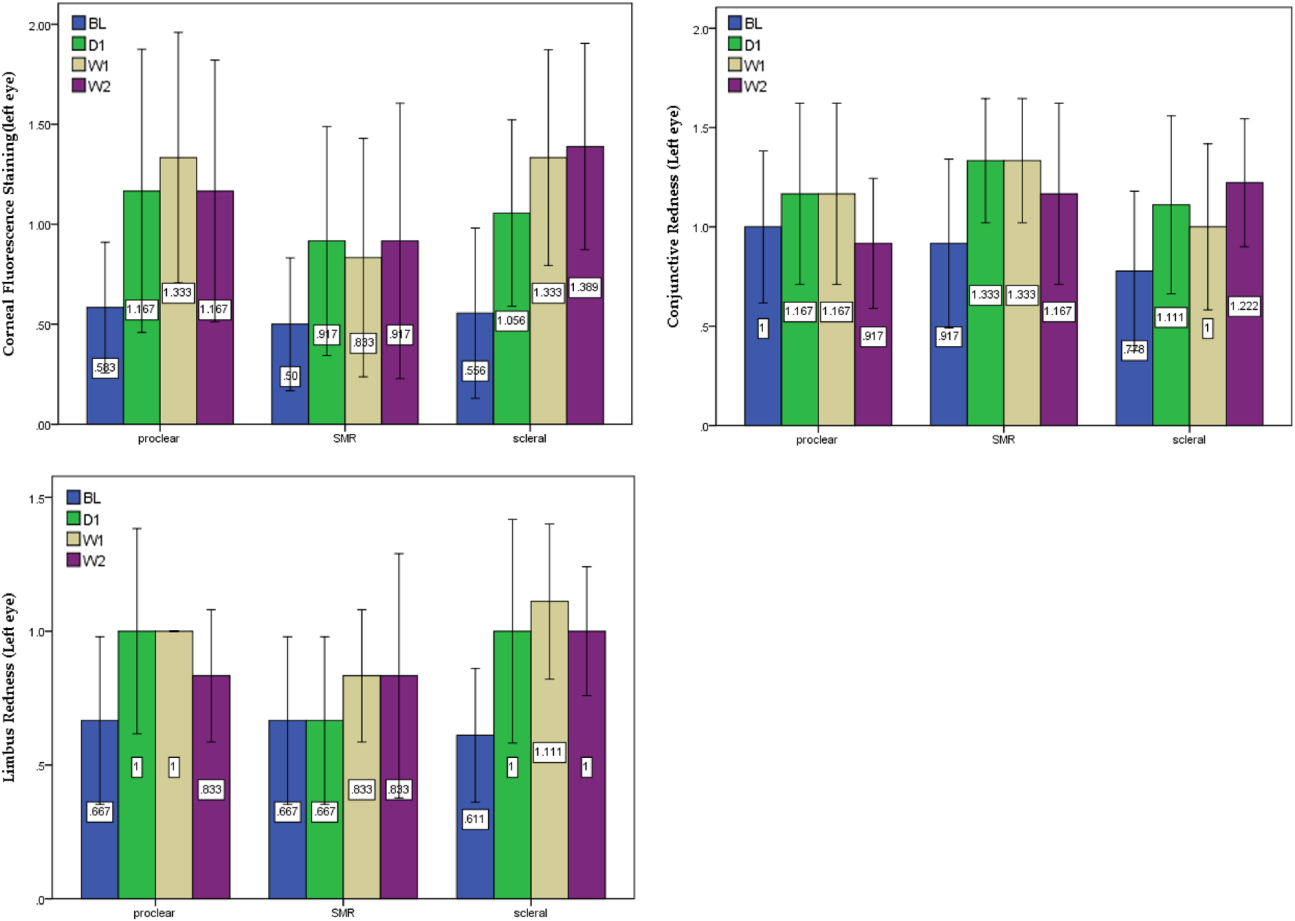
Ocular surface with corneal staining, conjunctival redness, and limbus redness between the three lenses and four times of measurement.

Two-way ANOVA, mixed design, and simple main effects were used to compare the differences between the three lenses and four times of measurement (Table 3 and Fig. 1). The results showed that ocular surface examination with different types of large-diameter multifocal contact lenses might influence the values of corneal fluorescence staining (*F* = 7.235, *p* < 0.000), conjunctival redness (*F* = 3.811, *p* = 0.012), and limbus redness (*F* = 3.443, *p* = 0.042). Simple main effects analysis indicated that all three types of largediameter multifocal contact lenses had negative impacts on the ocular surface, of which the Optimum RGPs lens had the greatest impact on corneal (*F* = 4.489, *p* = 0.007) and limbus (*F* = 3.357, *p* = 0.026); post hoc analysis showed significant differences between baseline, week 1, and week 2. Additionally, no significant was observed between different materials (soft and rigid) and optical (CN and CD) designs, it appeared that the lens diameter might be the sole factor affecting the ocular surface, the larger the contact lens diameter, the more redness reaction was shown on the cornea and limbus.

**Table 3:** Two-way ANOVA, mixed design, and simple main effects analysis on ocular surface measurement.

	Two-way ANOVA	Main effects	Measurement		Post hoc(* p* < 0.05)
			F	*p*	
Corneal Fluorescence Staining (Left eye)	*F* = 7.235, *p* = 0.001**	Proclear:	2.209	.106	
		SMR	1.582	.212	
		Optimum:	4.489	.007	BL > W1, BL > W2
Conjunctive Redness (Left eye)	*F* = 3.811 *p* = 0.012*	Proclear:	1.320	.284	
		SMR	3.163	.079	
		Optimum:	2.439	.075	
Limbus Redness (Left eye)	*F* = 3.443 *p* = 0.042*	Proclear:	1.984	.136	
		SMR	1.375	.268	
		Optimum:	3.357	.026	BL > W1, BL > W2

* p < .05, ** *p* < .01, > means “better than”.

### Tear quality

#### Tear changes when wearing contact lenses (Lens-on, tears on the front surface of the lens)

**Figure 2:**
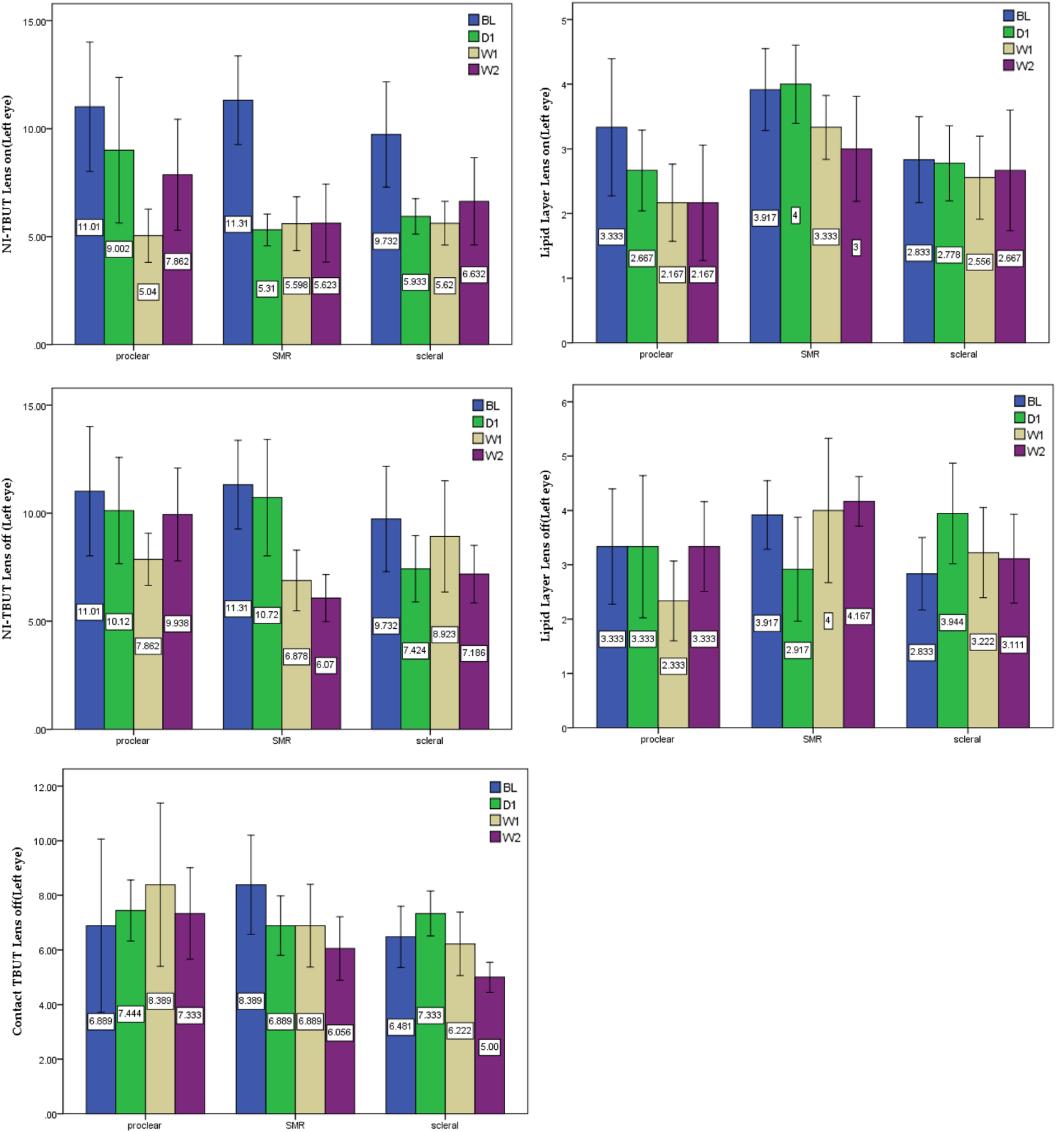
Tear changes of NI-TBUT, lipid-layer thickness, and Contact-TBUT between the three lenses and four times of measurement.

Two-way ANOVA, mixed design, and simple main effects were used to compare the differences between the three lenses and four times of measurement (Table 4 and Fig. 2). The results showed that the values of non-contact tear breakup time (NI-TBUT) (*F* = 19.421, *p* < 0.001) and lipid-layer thickness (*F* = 5.222, *p* = 0.002) became significantly worse when wearing contact lenses (Lens-on). Further analysis of the main effects comparison revealed that each type of large diameter contact lenses had significant impacts on NI-TBUT (Proclear: *F* = 6.177, *p* = 0.002, SMR: *F* = 17.132, *p* < 0.001, Optimum: *F* = 4.807, *p* = 0.005) and lipid layer (Proclear: *F* = 7.118, *p* = 0.001, SMR: *F* = 3.778, *p* = 0.020, Optimum: *F* = 0.291, *p* = 0.832), of which Optimum lens did not differ from the baseline, indicating that Optimum lens had little impact on lipid layer while wearing. The above comparison showed that wearing contact lenses affected the NI-TBUT and the lipid-layer thickness on the front surface of the lens with wearing duration. Additionally, no significant was observed between different materials (soft and rigid) and optical (CN and CD) designs, it appeared that the lens diameter might be the main factor affecting the tear quality while lens on. Optimum lens exerts the greatest positive influence and shows a more stable trend compared with the other two lenses.

**Table 4:** Two-way ANOVA, mixed design, and simple main effects analysis on tear quality measurement.

	Two-way ANOVA	Main effects	Measurement		Post hoc(* p* < 0.05)
			F	*p*	
NI-TBUT (Lens on_Left eye)	*F* = 19.421 *p* < 0.001**	Proclear:	6.177	.002	BL > W1
		SMR	17.132	< .001	BL > D1, BL > W1, BL > W2
		Optimum:	4.807	.005	BL > D1
Lipid Layer (Lens on_Left eye)	*F* = 5.222 *p* = 0.002**	Proclear:	7.118	.001	BL > W1, BL > W2
		SMR	3.778	.020	BL > W2, D1 > W2
		Optimum:	0.291	.832	
NI-TBUT (Lens off_Left eye)	*F* = 7.965 *p* < 0.001**	Proclear:	2.836	.094	
		SMR	11.786	< .001	BL > W1, BL > W2, D1 > W2
		Optimum:	2.017	.123	
Lipid Layer (Lens off_Left eye)	*F* = 3.811 *p* = 0.012*	Proclear:	1.759	.174	
		SMR	8.423	< .001	W2 > D1
		Optimum:	7.043	< .001	D1 > BL
Contact-TBUT (Lens off_Left eye)	*F* = 3.443 *p* = 0.042*	Proclear:	0.298	.827	
		SMR	2.491	.050	BL > W2
		Optimum:	4.860	.005	D1 > W2
NI-TBUT (Lens on_Left eye)	*F* = 19.421 *p* < 0.001**	Proclear:	6.177	.002	BL > W1
		SMR	17.132	< .001	BL > D1, BL > W1, BL > W2
		Optimum:	4.807	.005	BL > D1
Lipid Layer (Lens on_Left eye)	*F* = 5.222 *p* = 0.002**	Proclear:	7.118	.001	BL > W1, BL > W2
		SMR	3.778	.020	BL > W2, D1 > W2
		Optimum:	0.291	.832	
NI-TBUT (Lens off_Left eye)	*F* = 7.965 *p* < 0.001**	Proclear:	2.836	.094	
		SMR	11.786	< .001	BL > W1, BL > W2, D1 > W2
		Optimum:	2.017	.123	
Lipid Layer (Lens off_Left eye)	*F* = 3.811 *p* = 0.012*	Proclear:	1.759	.174	
		SMR	8.423	< .001	W2 > D1
		Optimum:	7.043	< .001	D1 > BL
Contact-TBUT (Lens off_Left eye)	*F* = 3.443 *p* = 0.042*	Proclear:	0.298	.827	
		SMR	2.491	.050	BL > W2
		Optimum:	4.860	.005	D1 > W2
NI-TBUT (Lens on_Left eye)	*F* = 19.421 *p* < 0.001**	Proclear:	6.177	.002	BL > W1
		SMR	17.132	< .001	BL > D1, BL > W1, BL > W2
		Optimum:	4.807	.005	BL > D1
Lipid Layer (Lens on_Left eye)	*F* = 5.222 *p* = 0.002**	Proclear:	7.118	.001	BL > W1, BL > W2
		SMR	3.778	.020	BL > W2, D1 > W2
		Optimum:	0.291	.832	
NI-TBUT (Lens off_Left eye)	*F* = 7.965 *p* < 0.001**	Proclear:	2.836	.094	
		SMR	11.786	< .001	BL > W1, BL > W2, D1 > W2
		Optimum:	2.017	.123	
Lipid Layer (Lens off_Left eye)	*F* = 3.811 *p* = 0.012*	Proclear:	1.759	.174	
		SMR	8.423	< .001	W2 > D1
		Optimum:	7.043	< .001	D1 > BL
Contact-TBUT (Lens off_Left eye)	*F* = 3.443 *p* = 0.042*	Proclear:	0.298	.827	
		SMR	2.491	.050	BL > W2
		Optimum:	4.860	.005	D1 > W2

* p < .05, ** *p* < .01, > means “better than”.

#### Tear changes after removing contact lenses (Lens-off, tears on the ocular surface).

Like tears on the front surface, analysis on the ocular surface indicated that NI-TBUT (*F* = 7.965, *p* < 0.001), lipid-layer thickness (*F* = 3.811, *p* = 0.012), and Contact-TBUT (*F* = 3.443, *p* = 0.042) were significantly different while removing contact lenses. Main effects comparison analysis of the three lens groups revealed that although the performance of tear breakup time for the three lens groups was still worse after the contact lenses were removed (NI-TBUT: Proclear: *F* = 2.836, *p* = 0.094; SMR: *F* = 11.786, *p* < 0.001; Optimum: *F* = 2.017, *p* = 0.123; Contact-TBUT: Proclear: *F* = 0.298, *p* = 0.827, SMR: *F* = 2.491, *p* = 0.050, Optimum: *F* = 4.860, *p* = 0.005), especially for the SMR lenses. What was surprising that the positive performance of lipid-layer thickness after removing contact lenses (Proclear: *F* = 1.759, *p* = 0.174, SMR: *F* = 8.423, *p* < 0.001, Optimum: *F* = 7.043, *p* < 0.001), especially shown on the SMR and Optimum lenses. This might suggest that larger-diameter or different material design (soft and rigid, *F* = 7.985, *p* < 0.001) contact lenses are beneficial to lipid-layer thickness.

### Visual quality

#### Blinking frequency

**Figure 3:**
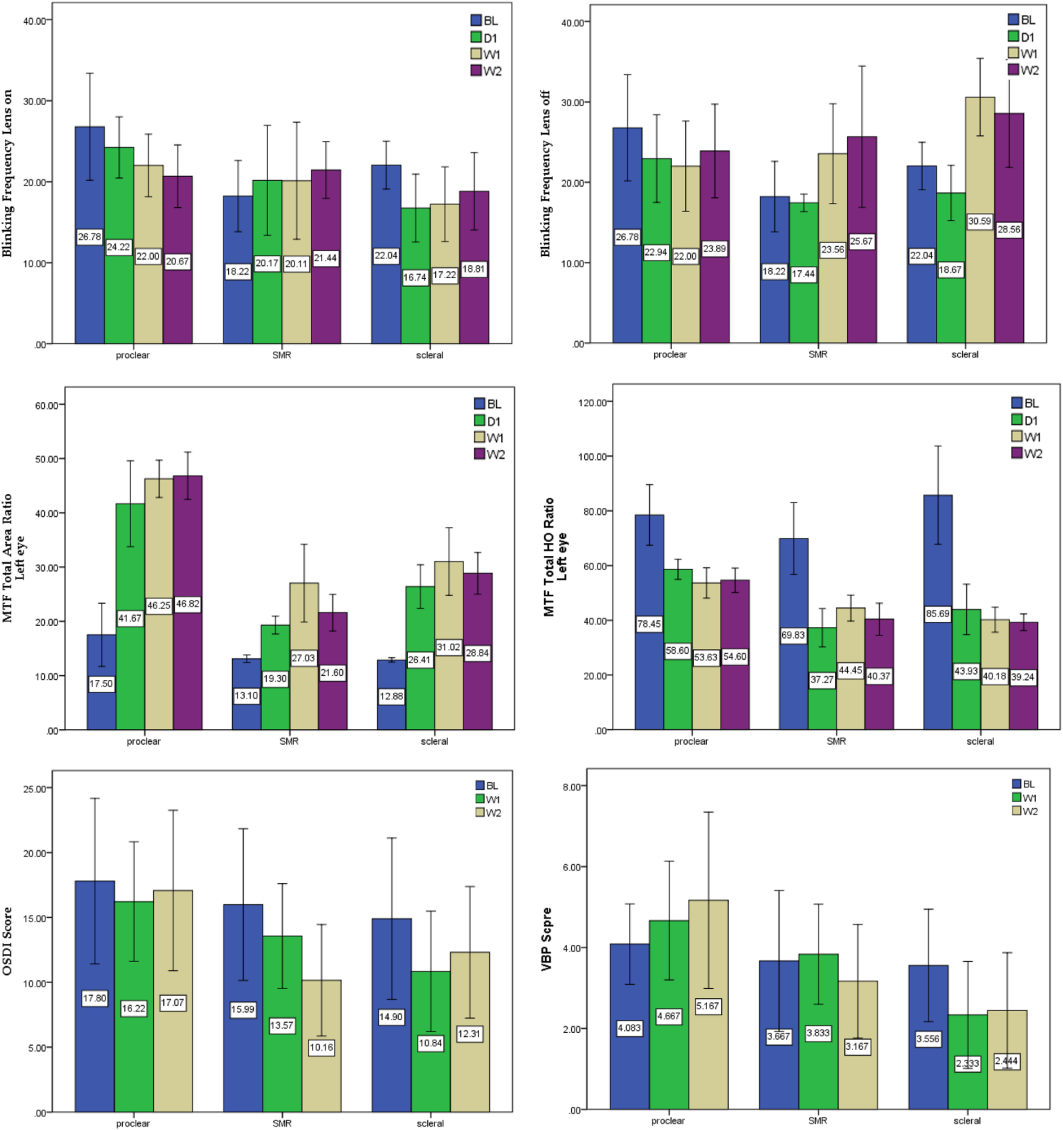
Visual quality between the three lenses and four times of measurement.

Table 5 and Fig. 3 showed that the blinking frequency while wearing contact lenses was relatively stable, and there was no significant difference compared to that when wearing spectacles (*F* = 1.088, *p* = 0.357). Even so, there were positive trends in the performance of Proclear and Optimum lenses (both were soft lens); however, upon removing contact lenses (*F* = 5.318, *p* = 0.002), the blinking frequency of Optimum (*F* = 7.729, *p* < 0.001) lenses increased significantly, indicating that the comfort of wearing large-diameter rigid contact lenses was relatively satisfactory.

**Table 5:** Two-way ANOVA, mixed design, and simple main effects analysis on visual quality measurement.

	Two-way ANOVA	main effects	Measurement		Post hoc(* p* < 0.05)
			F	*p*	
Blinking Frequency Lens on	*F* = 1.088 *p* = 0.357	Proclear:	1.899	.149	
		SMR	0.421	.739	
		Optimum:	2.198	.100	
Blinking Frequency Lens off	*F* = 5.318 *p* = 0.002**	Proclear:	0.895	.454	
		SMR	3.050	.057	
		Optimum:	7.729	< .001	W1 > BL , W1 > D1
MTF Total Area Ratio (Left eye)	*F* = 78.958 *p* < 0.001**	Proclear:	71.318	< .001	D1 > BL, W1 > BL, W2 > BL
		SMR	13.752	< .001	D1 > BL, W1 > BL, W2 > BL
		Optimum:	20.766	< .001	D1 > BL, W1 > BL, W2 > BL
MTF Total HO Ratio (Left eye)	*F* = 39.467 *p* < 0.001**	Proclear:	13.440	< .001	BL > D1, BL > W1, BL > W2
		SMR	19.702	< .001	BL > D1, BL > W1, BL > W2
		Optimum:	20.211	< .001	BL > D1, BL > W1, BL > W2
OSDI Score (BL, W1, W2)	*F* = 3.443 *p* = 0.042*	Proclear:	0.207	.814	
		SMR	8.117	.002	W2 > BL, W2 > W1
		Optimum:	3.567	.041	W1 > BL,
VBP Score (BL, W1, W2)	*F* = 19.421 *p* < 0.001**	Proclear:	1.045	.369	
		SMR	1.233	.311	
		Optimum:	3.132	.047	W1 > BL, W2 > BL

* p < .05, ** *p* < .01, > means “better than”.

#### Modulation transfer function

The higher the contrast of the modulation transfer function (MTF), the better the resolution. After the correction of the total area ratio by the contact lens, compared with unaided vision, the resolution of the three contact lenses improved significantly (*F* = 78.958, *p* < 0.001). Main effects comparison analysis revealed that day 1, week 1, and week 2 performed better than baseline in each lens group (Table 5 and Fig. 3, Proclear: *F* = 71.318, *p* < 0.001; SMR: *F* = 13.752, *p* < 0.001; Optimum: *F* = 20.766, *p* < 0.001).

Additionally, the performance of the MTF total higher order (HO) ratio was significantly worse (*F* = 39.467, *p* < 0.001). Main effects comparison analysis revealed that the performance was worse on day 1, week 1, and week 2 compared with baseline in each lens group (Table 5 and Fig. 3, Proclear: *F* = 13.440,* p* < 0.001, SMR: *F* = 19.702, *p* < 0.001, Optimum: *F* = 20.211,* p* < 0.001). This might be related to the design of the lens, as Poclear lens is CN and had a smaller effect on the MTF total HO ratio compared with the effect of the distance-center design of the SMR and Optimum lenses which are CD; however, performance on day 1, week 1, and week 2 was significantly worse in each lens group compared with the asymptotic multifocal design.

#### Questionnaire

The OSDI questionnaires were performed at three different times: baseline, after contact lenses were worn for 1 week, and after contact lenses were worn for 2 weeks. There was significant variation among the time points (*F* = 3.443, *p* = 0.042), with subjects reporting higher satisfaction with SMR (*F* = 8.117, *p* = 0.002) and Optimum (*F* = 3.567, *p* = 0.041) after 1 or 2 weeks of use. Additionally, people with dry eye or discomfort reported increased comfort after wearing the lenses. Visual Behavior Performance scores also significantly improved (*F* = 19.421, *p* < 0.001), particularly in association with the Optimum lens (*F* = 3.132, *p* = 0.047) after 1 or 2 weeks of use. This suggests that large-diameter contact lenses might be helpful for near work, comfort, perception, and balance.

### Visual function

**Figure 4:**
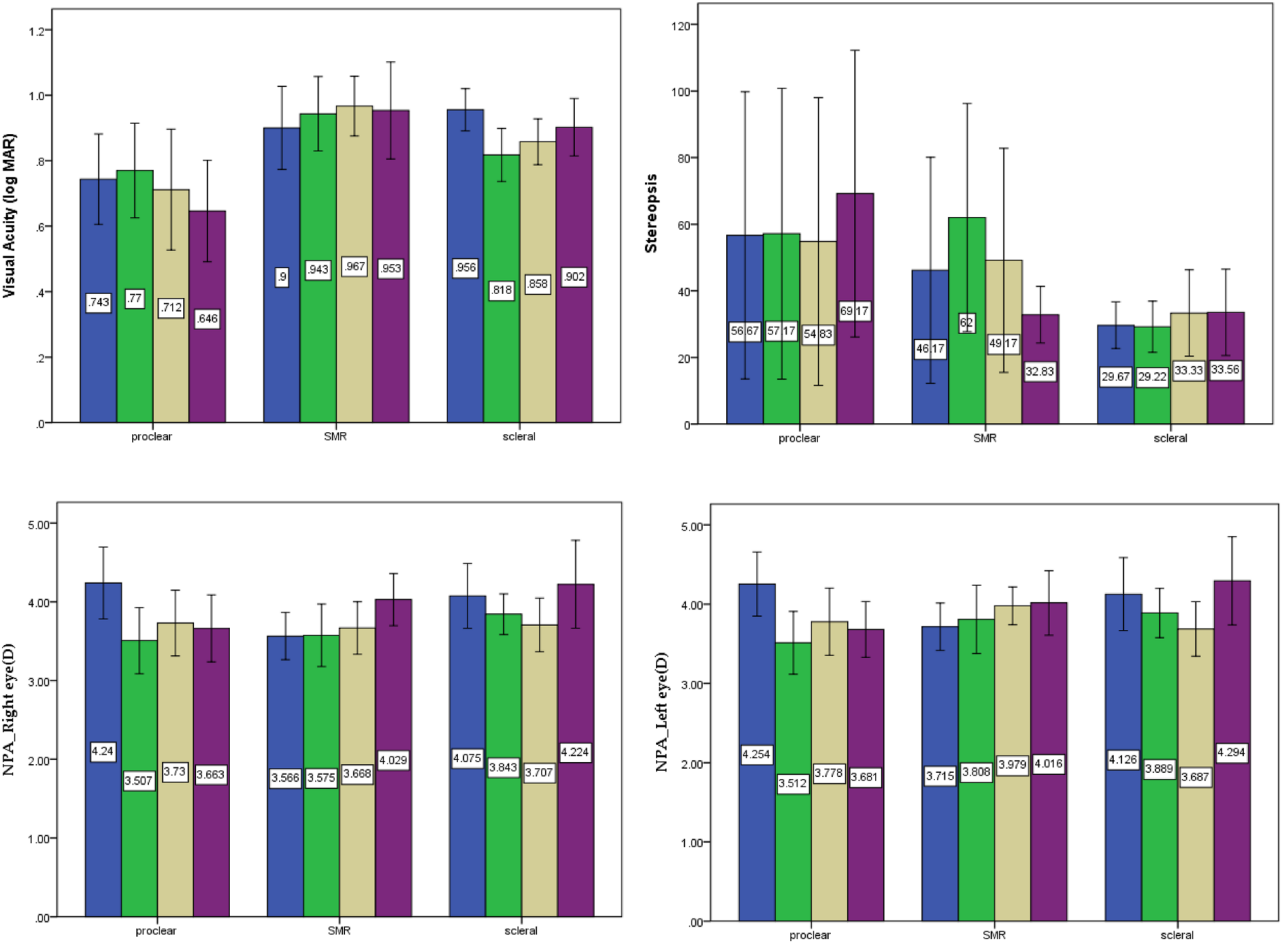
Visual Function between the three lenses and four times of measurement.

The two-way ANOVA analysis of near visual acuity (*F* = 0.461, *p* = *0.637*) and stereopsis (*F* = 0.440, *p* = *0.648*) did not show any significant difference between the three lens groups or the four times points of measurement (Table 6 and Fig. 4). However, there was an effect on near visual acuity. The Optimum lenses performed better than spectacles for correcting near visual acuity (*F* = 3.847, *p* = *0.015*). Additionally, Proclear lens performed significant worse than baseline both on the right and left eyes in terms of the near point of accommodation (right eye: *F* = 3.358, *p* = 0.021; left eye: *F* = 5.377,* p* = 0.002). Nevertheless, there was no significant difference between SMR and Optimum, and there was even a positive trend after baseline in terms of the near point of accommodation.

**Table 6:** Two-way ANOVA, mixed design, and simple main effects analysis on visual function examination.

	Two-way ANOVA	Main effects	Measurement		Post hoc (* p* < 0.05)
			F	*p*	
Near Visual Acuity	*F* = 0.461 *p* = *0*.637	Proclear:	1.656	.196	
		SMR	.844	.480	
		Optimum:	3.847	.015	D1 > BL
Stereopsis	*F* = 0.440 *p* = *0*.648	Proclear:	3.633	.076	
		SMR	2.904	.092	
		Optimum:	0.486	.694	
Near Point of Accommodation (D)—Right eye	F = 3.358 *p* = *0*.021*	Proclear:	12.255	< .001	BL > D1, BL > W1, BL > W2
		SMR	1.048	.384	
		Optimum:	3.283	.078	
Near Point of Accommodation (D)— Left eye	F = 5.377 *p* = *0*.002**	Proclear:	13.436	< .001	BL > D1, BL > W1, BL > W2
		SMR	2.810	.081	
		Optimum:	3.007	.074	

* p < .05, ** *p* < .01, > means “better than”.

## Discussion

Analysis of the ocular surface condition after wearing large-diameter multifocal contact lenses for 2 weeks revealed that although the redness of the limbus and corneal fluorescent staining worsened compared to baseline measurements, but the values were both within the clinically normal and acceptable standard^38^. For people with cornea damage, previous research has indicated that wearing large-diameter rigid contact lenses can significantly reduce corneal fluorescent staining^29^. The present study did not demonstrate any significant improvements in the state of the ocular surfaces, which may be related to the health of the initial eye itself. For patients who wear contact lenses to prevent ocular surface damage, it may be better to assess any effect after utilizing those lenses for extended period of time.

Measurements of tear quality can be conducted in two stages: the front surface of the contact lens and the front surface of the cornea^39^. The tears on the front surface of both the contact lens and the cornea are affected by lenses usage. After 1 week of wearing the contact lens, both the NI-TBUT and lipid-layer thickness significantly decreased. The correlation between the two variables may be used to evaluate DES caused by contact lenses^40–42^. Previous studies have also pointed out that the hydrophobic area of the lens will increase tear evaporation and reduce tear breakup time on the non-moisturizing lens surface. In addition, the results may be due to the electrostatic effect between the lipid layer and the contact lenses, resulting in oil deposition on the lens, which decreases the lipid-layer thickness and increases tear evaporation^40^. affecting the integrity of tear coverage.

One limiting factor of this study was the duration being only 2 weeks of data collection for each subject. Given the limited time frame, it is impossible to conclude whether prolonged wear of the lens beyond the 2-week period will have continuous negative impact on the tear film quality the front of the contact lens and the front surface of the cornea. However, analysis of tear changes showed that among the three lens groups, Optimum exerts higher stability of tear films, which may be related to the design and rigid lens material. Further investigation is necessary to be conclusive.

Blink frequency is a major factor affecting the stability of the tear film. Each complete blink will allow even distribution of the tear film covering the ocular surface to increase stability and concurrently improve visual quality^43^. The analysis results of blink frequency showed no significant change amont the three groups while wearing the lenses. However, there is significant improvement for the Optimum group after lens removal. Therefore, it is speculated the factors responsible for the change in tear balance are not affected by blink frequency, but rather conservation of ocular surface with adequate tear support.

The simulated value of the MTF of the total aberration and measurement results with contact lenses have better visual quality than the eye without wearing contact lenses at the beginning. This result shows that the difference is possibly due to the correction of ametropia^44^. In addition, material, design, and adaptation of contact lenses also affect changes in aberrations. Previous studies compared the performance of contrast sensitivity of spectacles, soft contact lenses, and rigid contact lenses. Although there was no significant difference among the three types of lenses, the contrast sensitivity of the rigid contact lenses was better than that of the soft contact lenses and spectacles. In addition, the rigid contact lens design with a large diameter can correct most of the corneal astigmatism and high-order aberrations^29,45^.

The subjective questionnaire showed little difference in the scores of the OSDI before and after wearing contact lenses. On the other hand, the visual behavior performance scale significantly improved after 1 week of lens wear. Previous studieshave proposed that there is a significant proportional relationship between the ocular surface disease index scale and the convergence fatigue symptom survey (CISS) showing benefits to enhance basic binocular visual function test to the evaluation of DES^46^. Although this portion is not entirely conclusive from the results of this subjective questionnaire, it still has reference value for the subjective comfort of wearing contact lenses. Visual function includes eye accommodation, stereo vision, and near visual acuity. The test results showed that after two weeks of lens wear, there was little difference in stereo vision compared to habitual spectacles correction. CD lens could induce better near visual acuity and accommodation; on the contrary, CN lens perform worse in accommodation^34,47–51^.

## Conclusions

Each lens design has its advantages and disadvantages. Patients can choose different modalities of presbyopia-correcting contact lenses based on their visual needs and adaptability. Although there may be specific negative impacts on the ocular surface and tear film with contact lens wear, one can still expect improvement in eye health, visual quality, and overall quality of life with proper lens fitting, wearing modality, and lens care. More studies need to be performed to explore a variety of contact lens designs that may be suitable for people with different needs and circumstances to provide more references on contact lens fitting, reduce complaints and discomfort from contact lens wear, and provide presbyopic adults with more choices to achieve comfortable vision and enhance quality of life.

## Data Availability

All data generated or analyzed during this study are included in this published article and its supplementary information files. Correspondence and requests for materials should be addressed to C.-Y.C.
